# Effects of established BMI-associated loci on obesity-related traits in a French representative population sample

**DOI:** 10.1186/1471-2156-15-62

**Published:** 2014-05-23

**Authors:** Louisa Goumidi, Dominique Cottel, Jean Dallongeville, Philippe Amouyel, Aline Meirhaeghe

**Affiliations:** 1 INSERM, U744; Institut Pasteur de Lille; Université Lille Nord de France, 1 rue du Pr. Calmette, BP 245, Lille Cedex F-59019, France

**Keywords:** Genetic predisposition score, Polymorphism, BMI, Obesity, General population

## Abstract

**Background:**

Genome-wide association studies have identified variants associated with obesity-related traits, such as the body mass index (BMI). We sought to determine how the combination of 31 validated, BMI-associated loci contributes to obesity- and diabetes-related traits in a French population sample. The MONA LISA Lille study (1578 participants, aged 35–74) constitutes a representative sample of the population living in Lille (northern France). Genetic variants were considered both individually and combined into a genetic predisposition score (GPS).

**Results:**

Individually, 25 of 31 SNPs showed directionally consistent effects on BMI. Four loci (*FTO*, *FANCL*, *MTIF3* and *NUDT3*) reached nominal significance (*p* ≤ 0.05) for their association with anthropometric traits. When considering the combined effect of the 31 SNPs, each additional risk allele of the GPS was significantly associated with an increment in the mean [95% CI] BMI of 0.13 [0.07-0.20] kg/m^2^ (*p* = 6.3x10^-5^) and a 3% increase in the risk of obesity (*p* = 0.047). The GPS explained 1% of the variance in the BMI. Furthermore, the GPS was associated with higher fasting glycaemia (*p* = 0.04), insulinaemia (*p* = 0.008), HbA1c levels (*p* = 0.01) and HOMA-IR scores (*p* = 0.0003) and a greater risk of type 2 diabetes (OR [95% CI] = 1.06 [1.00-1.11], *p* = 0.03). However, these associations were no longer statistically significant after adjustment for BMI.

**Conclusion:**

Our results show that the GPS was associated with a higher BMI and an insulin-resistant state (mediated by BMI) in a population in northern France.

## Background

According to the World Health Organization (WHO)’s criterion for obesity (body mass index (BMI) ≥ 30 kg/m^2^), up to 15% of the adults in Europe are obese [1]. The prevalence of obesity has more or less doubled since 1980 [2]. Obesity is a serious public health issue worldwide. Indeed, there is a well-documented relationship between a high BMI on one hand and mortality and morbidity due to chronic diseases (such as cardiovascular disease, certain cancers, type 2 diabetes (T2D) and osteoarthritis) on the other [3]. Accordingly, the WHO has declared obesity to be a global epidemic that affects both industrialized and non-industrialized countries [4].

Body fat mass is influenced by the combination of genetic factors and lifestyle factors (such as diet and physical activity). Family and twin studies have shown that genetic factors account for 40–70% of the population variation in BMI [5,6]; this may explain why people are not all equally affected by obesity in an obesogenic environment [7].

Genome-wide association studies (GWASs) have sought to elucidate the genetic basis of obesity and its related traits. To date, 32 genetic loci have been unequivocally associated with BMI [8]. Several studies have replicated these associations and have taken account of the combined impact of these GWAS-validated loci when considering BMI and other obesity-related phenotypes [8,9]. The objective of the present study was to replicate the combined effects of the established BMI-associated loci on BMI, body fat percentage, waist circumference, waist-to-hip ratio (WHR) and obesity risk in a representative sample of the general population in northern France (n = 1578). Furthermore, the high observed burden of obesity-related co-morbidities (such as insulin resistance and T2D) prompted us to test the impact of the BMI-associated loci on glucose-related traits and the risk of T2D.

## Results

### Characteristics of the MONA LISA Lille study

The characteristics of the study participants are summarized in Additional file 1: Table S1. Of the 1578 individuals, 37.3% were overweight, 22.4% were obese, and 9.2% had T2D. We selected 32 single nucleotide polymorphisms (SNPs) in or near the genes listed hereafter and that are known to be associated with BMI: *FTO, MC4R, TMEM18, GNPDA2, BDNF, NEGR1, SH2B1, ETV5, MTCH2, KCTD15, TFAP2B, NRXN3, FAIM2, SEC16B, RBJ-ADCY3-POMC, GPRC5B, MAP2K5-LBXCOR1, QPCTL-GIPR, TNNI3K, SLC39A8, FLJ35779-HMGCR, LRRN6C, TMEM160, FANCL, CADM2, PRKD1, LRP1B, PTBP2, MTIF3-GTF3A, RPL27A-TUB, NUDT3-HMGA1* and *ZNF608 *[8]. Genotyping of the *ZNF608* rs4836133 SNP failed and so the remaining 31 SNPs were investigated further.

### Single-variant analyses

All SNPs conformed to Hardy-Weinberg equilibrium (Additional file 1: Table S2). As the sample size was relatively small and the statistical power limited, no SNPs were significantly associated with any of the anthropometric parameters. Only the *FTO* rs9939609 and *FANCL* rs887912 SNPs were nominally associated with BMI (β ± SE = 0.49 ± 0.19 kg/m^2^, *p* = 0.008 and β ± SE = 0.54 ± 0.19 kg/m^2^, *p* = 0.005, respectively). Of the 31 tested SNPs, 25 were directionally consistent with the results reported in the original GWAS on BMI (Additional file 1: Table S3). This number was higher than that expected by chance (*p* = 0.0003 in a binomial test). Some SNPs were nominally associated with continuous anthropometric traits other than BMI (such as body fat percentage and hip and waist circumferences). The *FTO* rs9939609 and *FANCL* rs887912 SNPs were nominally associated with body fat percentage (β ± SE = 0.53 ± 0.27%, *p* = 0.05 and β ± SE = 0.69 ± 0.28%, *p* = 0.01, respectively). We also observed nominal associations between the *FANCL* rs887912 SNP and waist and hip circumferences (β ± SE = 1.03 ± 0.50 cm, *p* = 0.04 and β ± SE = 0.89 ± 0.39 cm, *p* = 0.02, respectively), between the *MTIF3* rs4771122 SNP and hip circumference (β ± SE = 0.84 ± 0.42 cm, *p* = 0.04) and between the *NUDT3* rs206936 SNP and WHR (β ± SE = -0.007 ± 0.003, *p* = 0.01).

### The genetic predisposition score, BMI and the obesity risk

The 31 SNPs were used to calculate a genetic predisposition score (GPS), which was normally distributed (mean: 27.7 ± 3.7 alleles; range: 13.8 to 38.9). We observed significant associations between the GPS and several anthropometric variables (such as BMI, body fat percentage, waist circumference and hip circumference; Table 1). The mean [95% confidence interval (CI)] allele effect of the GPS was +0.13 [0.07-0.20] kg/m^2^ (*p* = 6.3x10^-5^) for BMI, +0.14 [0.05-0.24]% (*p* = 0.004) for body fat percentage, +0.28 [0.11-0.45] cm (*p* = 0.001) for waist circumference and +0.24 [0.11-0.37] cm (*p* = 3.7x10^-4^) for hip circumference. We did not detect a statistically significant association between the GPS and WHR.

**Table 1:** Effect of the genetic predisposition score on anthropometric variables in the MONA LISA Lille study (n = 1546)

**Parameter**	**β**	**SE**	**LCL**	**UCL**	***p***^***1***^	***p***^***2***^
BMI (kg/m^2^)	0.13	0.03	0.07	0.20	6.3x10^-5^	-
Body fat (%)	0.14	0.05	0.05	0.24	0.004	-
Waist (cm)	0.28	0.09	0.11	0.45	0.001	0.65
Hip (cm)	0.24	0.07	0.11	0.37	3.7x10^-4^	0.74
Waist-to-hip ratio	0.0006	0.0004	-0.0003	0.0015	0.19	0.41

The β coefficients represent the effect sizes. SE: standard error. LCL: lower confidence limit; UCL: upper confidence limit.

^*1*^*p* values were adjusted for age, gender, physical activity, smoking status and alcohol consumption. ^*2*^*p* values were adjusted for age, gender, physical activity, smoking status, alcohol consumption and BMI.

Similar results were obtained after taking into account missing genotypes (Additional file 1: Table S4). Associations between the GPS and the waist and hip circumferences disappeared after further adjustment for BMI.

We also investigated the possible effect of interactions between the GPS and gender, physical activity (PA), smoking status and alcohol consumption on anthropometric variables but did not detect any significant interactions (data not shown).

To distinguish between the effects of the GPS and the effects of the covariables classically associated with BMI (age, gender, PA, smoking status and alcohol consumption), we compared the crude and adjusted models (Table 2). The GPS alone accounted for 1% of the variance in the BMI, whereas the covariables accounted for 6%. Overall, the GPS and the covariables explained 7% of the variance in the BMI.

**Table 2:** Effects of the crude and adjusted genetic predisposition score on BMI in the MONA LISA Lille study (n = 1546)

**Models**	**β**	**SE**	**LCL**	**UCL**	***p***	**Explained variance (%)**
Model 1	0.14	0.03	0.07	0.20	5.4x10^-5^	1.0
Model 2	0.13	0.03	0.07	0.2	6.3x10^-5^	7.0

The β coefficients represent the effect sizes. SE: standard error. LCL: lower confidence limit; UCL: upper confidence limit.

Model 1: crude *p* value. Model 2: *p* value adjusted for age, gender, physical activity, smoking status and alcohol consumption.

We also investigated the association between the GPS and the obesity risk. Each additional BMI-raising allele was associated with a 3% increase in the obesity risk (OR [95% CI] = 1.03 [1.00-1.07]; *p* = 0.047).

### The genetic predisposition score, glucose-related traits and the type 2 diabetes risk

Given that obesity is an important determinant of glycaemic traits and insulin resistance, we assessed the association between the GPS on one hand and fasting plasma glucose, HbA1c and insulin levels, the HOMA-IR and HOMA-B scores and the risk of T2D on the other. We detected significant associations between the GPS and higher fasting plasma glucose (β ± SE = +0.017 ± 0.008 mmol/L, *p* = 0.04), insulin (β ± SE = +0.14 ± 0.06 μIU/mL*, p* = 0.008) and HbA1c levels (β ± SE = +0.012 ± 0.005%, *p* = 0.01) and a higher HOMA-IR (β ± SE = +0.06 ± 0.02, *p* = 0.0003) (Table 3). The GPS was also significantly associated with a higher risk of T2D (adjusted OR [95%CI] = 1.06 [1.00-1.11], *p* = 0.03). However, these associations were no longer statistically significant after adjustment for BMI.

**Table 3:** Associations between the genetic predisposition score and glucose-related variables in the MONA LISA Lille study

	**β**	**SE**	**LCL**	**UCL**	**Model 1**	**Model 2**
					***p***	***p***
Fasting glucose (mmol/L)	0.017	0.008	0.001	0.033	0.04	0.35
Fasting insulin (μIU/mL)	0.14	0.06	0.03	0.24	0.008	0.46
HbA1c (%)	0.012	0.005	0.003	0.021	0.01	0.10
HOMA-IR	0.06	0.02	0.03	0.10	0.0003	0.10
HOMA-B	1.17	0.63	-0.07	2.42	0.18	0.83

The β coefficients represent the effect sizes. SE: standard error. LCL: lower confidence limit; UCL: upper confidence limit.

Model 1: values were adjusted for age, gender, physical activity, smoking status and alcohol consumption.

Model 2: values were adjusted for age, gender, physical activity, smoking status, alcohol consumption and BMI.

## Discussion

Although the MONA LISA Lille study’s statistical power was too low (68%) to detect significant individual associations, 25 of the 31 investigated SNPs presented effects with the expected direction. Moreover, the effect alleles for the *FTO* rs9939609 and *FANCL* rs887912 SNPs were nominally associated with higher BMI. The GPS (corresponding to the cumulative contribution of the 31 validated BMI-associated SNPs) showed a significant, positive association with BMI. Each additional effect allele was associated with a mean increment of 0.13 kg/m^2^ in the BMI (which corresponds to a weight increment of 376 g for a person measuring 1.70 m in height) and a 3% increase in the risk of obesity. The GPS was also significantly associated with body fat percentage and waist and hip circumferences, although the last two associations did not resist adjustment for BMI (suggesting that they were driven by overall general adiposity). The genetic susceptibility associated with the GPS explained only 1% of the variance in the BMI, whereas the combined effect of known lifestyle factors accounted for 6%. Although it is clear that (i) genetic factors account for 40–70% of the population variation in BMI and (ii) the 31 SNPs studied here have been robustly validated as BMI-susceptible variants in GWASs and replication studies, the SNPs’ combined effect on BMI and the obesity risk was quite small. However, our results are in agreement with previous reports [8,10,11].

Gene-environment interactions may also account for variance in the BMI. Several studies have reported that PA is associated with a reduction in the GPS’s impact on BMI [12,13]. Like others [12], we failed to detect significant interactions between the GPS and PA when considering several anthropometric traits (BMI, body fat percentage, waist and hip circumferences and WHR). Our failure to detect this interaction is probably due to the relatively small sample size. In fact, very large sample sizes are needed when exploring this type of interaction. For example, Ahmad *et al.* showed that a population size of 20,000 is required to detect a β_GE_ interaction effect of -0.07 kg/m^2 ^[13].

Given that obesity is a major risk factor for insulin resistance [14], the accumulation of obesity risk alleles may alter glucose metabolism and predispose the individual to T2D. To evaluate this hypothesis, we looked at whether the GPS was associated with glucose-related variables and the T2D risk in the MONA LISA Lille study. Indeed, we found significant associations between the GPS on one hand and higher fasting plasma glucose, insulin and HbA1c levels and insulin resistance on the other. We also showed that each additional BMI-raising allele was associated with a 6% increment in the T2D risk. Our results in a general population sample are consistent with previous reports. In a French case–control study, each additional allele in the GPS was associated with higher insulin resistance and a 3% increase in the T2D risk [15]. In the EPIC prospective cohort study, each additional allele in the GPS was also associated with a 4% increase in the T2D risk [10]. In both these previous studies (as in the present study), all the statistically significant associations were abolished after adjustment for BMI - meaning that overall general adiposity explained the association between the GPS and insulin resistance or T2D.

## Conclusions

Our results showed that the combination of common genetic variants was moderately associated with BMI and BMI-related variables in a sample of the general population from northern France. Despite the fact that the heritability of BMI is estimated to be 40-70% [5], the combination of 31 validated, BMI-associated loci only explained only 1% of the variance in the BMI (i.e. less than 2-4% of the heritability) [8]. Hence, characterization of this unexplained heritability requires other approaches.

## Methods

### The MONA LISA Lille study

The MONA LISA (Monitoring National du Risque Artériel; National Monitoring of Arterial Risk) Lille study was a population-based, cross-sectional study of a representative sample of 1578 participants recruited from within the Lille urban area in northern France. In accordance with the French legislation on biomedical research, the study protocol was approved by the appropriate independent ethics committee (Comité Consultatif de Protection des Personnes dans la Recherche Biomédicale de Lille) and written informed consent was obtained from all participants. The study design and methods are described in the Additional file 1: methods. Briefly, anthropometric traits were recorded during a physical examination of each individual and a blood sample was collected (for DNA extraction and clinical biochemistry assays). The BMI was calculated according to the Quetelet equation. Obesity was defined as a BMI of 30 kg/m^2^ or more. Type 2 diabetes was defined according to the 1997 American Diabetes Association definition (fasting plasma glucose ≥ 7.0 mmol/l and/or treatment for diabetes, including diet and/or oral antidiabetic drugs and/or insulin) [16].

### Genotyping

Single nucleotide polymorphisms were genotyped using KASPar technology (KBioscience, Hoddesdon, UK). The genotyping success rates ranged from 98.1% to 99.6%.

### Statistical analysis

Statistical analyses were performed with SAS 9.1 software (SAS Institute Inc., Cary, NC, USA).

The Hardy-Weinberg equilibrium was tested using a χ^2^ test with one degree of freedom.

The GPS was derived as described previously [17]. Briefly, a weighting method was used to calculate the GPS on the basis of 31 SNPs. Each SNP was weighted according to its relative effect size (i.e. the β coefficient). In order to measure the effect of each SNP on BMI with greater accuracy and precision, β coefficients were derived as described by Speliotes *et al. *[8]. We rescaled the weighted scores to reflect the number of risk alleles. Hence, each point on the GPS corresponded to one risk allele. When calculating the GPS, missing genotype data were replaced with the average allele count for the corresponding SNPs. However, individuals with missing genotypes for more than 10% of the loci were excluded from the GPS analyses (n = 30).

We used general linear regression models to test the associations of individual BMI-related SNPs and the GPS with adiposity-related traits (including BMI, body fat percentage, WHR, waist circumference and hip circumference) and glucose-related traits (assuming an additive effect of the BMI-increasing alleles). A logistic regression model was used to test the association between the GPS and the risk of obesity or T2D. Interactions between the GPS on one hand and gender, PA, smoking status and alcohol consumption on the other were tested by including the GPS, interaction variables and the interaction terms (GPS x interaction variables) in general linear regression models.

The associations between genetic variants and BMI, obesity and interactions were adjusted for age, gender, smoking status, PA and alcohol consumption. The associations between genetic variants and body fat percentage, WHR, waist circumference and hip circumference were adjusted for age, gender, smoking status, PA, and alcohol consumption including or not BMI, depending of models. The associations between genetic variants and biological parameters and the T2D risk were adjusted for age, gender, BMI, smoking status, PA and alcohol consumption. Data distributions for plasma glucose and insulin levels and HOMA-IR and HOMA-B scores were normalized by log transformation.

Bonferroni correction was used to adjust for the Hardy-Weinberg equilibrium and for the multiple testing in the individual obesity-related trait analyses. The threshold for statistical significance was set to *p* ≤ 0.0016 (for 31 independent SNPs). Nominal significance was defined as 0.0016 < *p* < 0.05.

For the GPS analyses, the threshold for statistical significance was set to *p* ≤ 0.05.

The power calculations for association analyses (performed *a priori* using Quanto v1.2.4 software (http://biostats.usc.edu/Quanto.html) on the basis of the mean BMI values from the MONA LISA Lille study and the effect allele frequencies and effect sizes originally reported by Speliotes *et al*. [8]) indicated that the statistical power of our study (for detecting a significant association between an individual SNP and BMI with a one-sided *p* value of 0.05) was 68%.

The power calculations for the GPS analysis were performed using the pwr package developed by Stéphane Champely. The statistical power for detecting significant association between GPS and BMI (using a *p* value at 0.05) was 99%.

## Abbreviations

BMI: Body mass index; CI: Confidence interval; GPS: Genetic predisposition score; GWAS: Genome-wide association study; OR: Odds ratio; PA: Physical activity; SE: Standard error; SNP: Single nucleotide polymorphism; T2D: Type 2 diabetes; WHR: Waist-to-hip ratio; WHO: World health organization.

## Competing interests

The authors declare that they have no competing interests.

## Authors’ contributions

DC, JD, PA, LG and AM designed the study and supervised the project. DC, JD and PA participated in the recruitment of participants. LG performed the statistical analyses. LG and AM interpreted the results. LG wrote the manuscript. LG and AM had primary responsibility for final content. All authors read and approved the final manuscript.

## Supplementary Material

Additional file 1: Table S1 Characteristics of the participants in the MONA LISA Lille study (n = 1578). **Table S2.** Genotype and allele distributions of the 31 successfully genotyped SNPs in the MONA LISA Lille study. **Table S3.** Associations between the 31 SNPs and the anthropometric variables in the MONA LISA Lille study (n = 1578). **Table S4.** Effect of the GPS on anthropometric variables in the MONA LISA Lille study for fully genotyped participants (n = 1326). **Methods.** The MONA LISA Lille study. Click here for file
