# Supplementary material for: Effects of established BMI-associated loci on obesity-related traits in a French representative population sample
Source: BMC Genet. 2014 May 23;15:62. doi: 10.1186/1471-2156-15-62 (PMC4035696; doi:10.1186/1471-2156-15-62)
Supplement: Additional file 1: Table S1 — Characteristics of the participants in the MONA LISA Lille study (n = 1578). Table S2. Genotype and allele distributions of the 31 successfully genotyped SNPs in the MONA LISA Lille study. Table S3. Associations between the 31 SNPs and the anthropometric variables in the MONA LISA Lille study (n = 1578). Table S4. Effect of the GPS on anthropometric variables in the MONA LISA Lille study for fully genotyped participants (n = 1326). Methods. The MONA LISA Lille study. [file 1471-2156-15-62-S1.doc]

**Additional file 1: Table S1.** Characteristics of the participants in the MONA LISA Lille study (n=1578).

| Age (yrs) |  | 55.4 ± 11.4 |
| --- | --- | --- |
| Men (%) |  | 49.6 |
|  |  |  |
| BMI (kg/m²) |  | 26.9 ± 5.0 |
| Body fat (%) |  | 31.2 ± 9.3 |
| Waist (cm) |  | 92.9 ± 14.2 |
| Hip (cm) |  | 104.6 ± 10.1 |
| Waist-to-hip ratio |  | 0.89 ± 0.08 |
|  |  |  |
| Fasting glucose (mmol/L) |  | 5.48 ± 1.22 |
| Fasting insulin (µIU/mL) |  | 11.54 ± 8.19 |
| HbA1c (%) |  | 2.92 ± 2.49 |
| HOMA-IR |  | 129.8 ± 90.5 |
| HOMA-B |  | 5.5 ± 0.7 |
|  |  |  |
| Overweight (%) |  | 37.3 |
| Obesity (%) |  | 22.4 |
| Type 2 diabetes (%) |  | 9.2 |
|  |  |  |

Data are expressed as the mean ± standard deviation or in %.

**Additional file 1: Table S2.** Genotype and allele distributions of the 31 successfully genotyped SNPs in the MONA LISA Lille study.

|  |  |  |  |  |  |  |  |  |  |  |
| --- | --- | --- | --- | --- | --- | --- | --- | --- | --- | --- |
| **Locus** | **SNP** |  | **EA/NEA** | **EAF/NEAF** |  |  | **Genotypes** |  |  | ***p* HWE** |
|  |  |  |  |  |  | **0** | **1** | **2** |  |  |
|  |  |  |  |  |  | **n (frequency)** | **n (frequency)** | **n (frequency)** |  |  |
|  |  |  |  |  |  |  |  |  |  |  |
| *FTO* | rs9939609 |  | A/T | 0.39/0.61 |  | 580 (0.37) | 756 (0.48) | 229 (0.15) |  | 0.49 |
| *TMEM18* | rs2867125 |  | C/T | 0.83/0.17 |  | 44 (0.03) | 432 (0.27) | 1094 (0.70) |  | 0.86 |
| *MC4R* | rs571312 |  | A/C | 0.23/0.77 |  | 944 (0.60) | 533 (0.34) | 93 (0.06) |  | 0.13 |
| *GNPDA2* | rs10938397 |  | G/A | 0.42/0.58 |  | 529 (0.34) | 762 (0.49) | 274 (0.17) |  | 0.99 |
| *BDNF* | rs10767664 |  | A/T | 0.77/0.23 |  | 95 (0.06) | 543 (0.35) | 928 (0.59) |  | 0.19 |
| *NEGR1* | rs2815752 |  | A/G | 0.63/0.37 |  | 202 (0.13) | 739 (0.48) | 607 (0.39) |  | 0.33 |
| *SH2B1* | rs7359397 |  | T/C | 0.36/0.64 |  | 641 (0.41) | 722 (0.46) | 202 (0.13) |  | 0.95 |
| *ETV5* | rs9816226 |  | T/A | 0.82/0.18 |  | 48 (0.03) | 461 (0.29) | 1060 (0.68) |  | 0.80 |
| *MTCH2* | rs3817334 |  | T/C | 0.39/0.61 |  | 591 (0.38) | 739 (0.47) | 240 (0.15) |  | 0.72 |
| *KCTD15* | rs29941 |  | G/A | 0.69/0.31 |  | 168 (0.11) | 636 (0.40) | 763 (0.49) |  | 0.04 |
| *SEC16B* | rs543874 |  | G/A | 0.18/0.82 |  | 1064 (0.68) | 461 (0.29) | 47 (0.03) |  | 0.73 |
| *TFAP2B* | rs987237 |  | G/A | 0.17/0.83 |  | 1082 (0.69) | 438 (0.28) | 45 (0.03) |  | 0.93 |
| *FAIM2* | rs7138803 |  | A/G | 0.36/0.64 |  | 631 (0.40) | 726 (0.47) | 208 (0.13) |  | 0.97 |
| *NRXN3* | rs10150332 |  | C/T | 0.20/0.80 |  | 994 (0.64) | 494 (0.32) | 69 (0.04) |  | 0.45 |
| *RBJ* | rs713586 |  | C/T | 0.45/0.55 |  | 484 (0.31) | 743 (0.47) | 338 (0.22) |  | 0.10 |
| *GPRC5B* | rs12444979 |  | C/T | 0.85/0.15 |  | 36 (0.02) | 389 (0.25) | 1143 (0.73) |  | 0.67 |
| *MAP2K5* | rs2241423 |  | G/A | 0.77/0.23 |  | 99 (0.06) | 528 (0.34) | 939 (0.60) |  | 0.04 |
| *QPCTL* | rs2287019 |  | C/T | 0.81/0.19 |  | 48 (0.03) | 503 (0.32) | 1015 (0.65) |  | 0.13 |
| *TNNI3K* | rs1514175 |  | A/G | 0.42/0.58 |  | 529 (0.34) | 761 (0.48) | 276 (0.18) |  | 0.94 |
| *SLC39A8* | rs13107325 |  | T/C | 0.06/0.94 |  | 1390 (0.88) | 174 (0.11) | 7 (0.01) |  | 0.54 |
| *FLJ35779* | rs2112347 |  | T/G | 0.65/0.35 |  | 195 (0.13) | 694 (0.44) | 678 (0.43) |  | 0.4 |
| *LRRN6C* | rs10968576 |  | G/A | 0.29/0.71 |  | 798 (0.51) | 623 (0.40) | 139 (0.09) |  | 0.27 |
| *TMEM160* | rs3810291 |  | A/G | 0.68/0.32 |  | 177 (0.11) | 665 (0.43) | 727 (0.46) |  | 0.18 |
| *FANCL* | rs887912 |  | T/C | 0.28/0.72 |  | 841 (0.53) | 590 (0.38) | 138 (0.09) |  | 0.02 |
| *CADM2* | rs13078807 |  | G/A | 0.20/0.80 |  | 988 (0.63) | 510 (0.33) | 65 (0.04) |  | 0.94 |
| *PRKD1* | rs11847697 |  | T/C | 0.04/0.96 |  | 1442 (0.917) | 122 (0.078) | 8 (0.005) |  | 0.003 |
| *LRP1B* | rs2890652 |  | C/T | 0.17/0.83 |  | 1083 (0.69) | 432 (0.27) | 56 (0.04) |  | 0.12 |
| *PTBP2* | rs1555543 |  | C/A | 0.57/0.43 |  | 274 (0.17) | 795 (0.51) | 500 (0.32) |  | 0.17 |
| *MTIF3* | rs4771122 |  | G/A | 0.25/0.75 |  | 888 (0.57) | 571 (0.37) | 100 (0.06) |  | 0.52 |
| *RPL27A* | rs4929949 |  | C/T | 0.49/0.51 |  | 428 (0.27) | 740 (0.47) | 400 (0.26) |  | 0.03 |
| *NUDT3* | rs206936 |  | G/A | 0.21/0.79 |  | 977 (0.63) | 520 (0.33) | 68 (0.04) |  | 0.91 |
|  |  |  |  |  |  |  |  |  |  |  |

EA: effect allele. NEA: non-effect allele. EAF: effect allele frequency. NEAF: non-effect allele frequency. HWE: Hardy-Weinberg Equilibrium. Genotypes were coded as 0/1/2, indicating the number of copies of the designated effect alleles per subject.

**Additional file 1: Table S3.** Associations between the 31 SNPs and the anthropometric variables in the MONA LISA Lille study (n=1578).

|  |  |  |  |  | | |  |  | | |  |  | | |  |  | | |  |  | | |
| --- | --- | --- | --- | --- | --- | --- | --- | --- | --- | --- | --- | --- | --- | --- | --- | --- | --- | --- | --- | --- | --- | --- |
|  |  |  |  | **BMI (kg/m²)** | | |  | **Body fat (%)** | | |  | **Waist (cm)** | | |  | **Hip (cm)** | | |  | **Waist-to-hip ratio** | | |
|  |  |  |  |  |  |  |  |  |  |  |  |  |  |  |  |  |  |  |  |  |  |  |
|  |  |  |  |  |  |  |  |  |  |  |  |  |  |  |  |  |  |  |  |  |  |  |
| ***Nearest gene*** | **SNP** | **EA** |  | **β** | **SE** | ***p*** |  | **β** | **SE** | ***p*** |  | **β** | **SE** | ***p*** |  | **β** | **SE** | ***p*** |  | **β** | **SE** | ***p*** |
|  |  |  |  |  |  |  |  |  |  |  |  |  |  |  |  |  |  |  |  |  |  |  |
| *FTO* | rs9939609 | A |  | 0.49 | 0.18 | **0.008** |  | 0.53 | 0.27 | **0.05** |  | 0.88 | 0.47 | 0.06 |  | 0.51 | 0.37 | 0.17 |  | 0.004 | 0.002 | 0.10 |
| *TMEM18* | rs2867125 | C |  | 0.25 | 0.24 | 0.3 |  | 0.27 | 0.35 | 0.44 |  | 0.2 | 0.62 | 0.75 |  | 0.64 | 0.49 | 0.19 |  | -0.003 | 0.003 | 0.27 |
| *MC4R* | rs571312 | A |  | 0.1 | 0.21 | 0.64 |  | 0.08 | 0.3 | 0.79 |  | 0.93 | 0.53 | 0.08 |  | 0.59 | 0.42 | 0.16 |  | 0.003 | 0.003 | 0.21 |
| *GNPDA2* | rs10938397 | G |  | 0.18 | 0.18 | 0.32 |  | 0.27 | 0.26 | 0.3 |  | 0.19 | 0.47 | 0.68 |  | 0.32 | 0.37 | 0.38 |  | -0.001 | 0.002 | 0.82 |
| *BDNF* | rs10767664 | A |  | 0.34 | 0.21 | 0.1 |  | 0.49 | 0.3 | 0.1 |  | 0.65 | 0.53 | 0.23 |  | 0.41 | 0.42 | 0.33 |  | 0.002 | 0.003 | 0.39 |
| *NEGR1* | rs2815752 | A |  | -0.11 | 0.19 | 0.57 |  | -0.25 | 0.27 | 0.36 |  | -0.57 | 0.48 | 0.24 |  | -0.64 | 0.38 | 0.09 |  | 0.000 | 0.002 | 0.91 |
| *SH2B1* | rs7359397 | T |  | 0.07 | 0.19 | 0.71 |  | -0.45 | 0.27 | 0.1 |  | -0.05 | 0.48 | 0.92 |  | 0.35 | 0.38 | 0.35 |  | -0.004 | 0.002 | 0.12 |
| *ETV5* | rs9816226 | T |  | -0.04 | 0.23 | 0.88 |  | -0.1 | 0.34 | 0.78 |  | 0.24 | 0.6 | 0.69 |  | 0.21 | 0.47 | 0.66 |  | 0.001 | 0.003 | 0.84 |
| *MTCH2* | rs3817334 | T |  | 0.03 | 0.18 | 0.87 |  | -0.14 | 0.26 | 0.6 |  | 0.11 | 0.47 | 0.81 |  | 0.14 | 0.37 | 0.71 |  | 0.000 | 0.002 | 0.93 |
| *KCTD15* | rs29941 | G |  | 0.14 | 0.19 | 0.44 |  | 0.2 | 0.27 | 0.46 |  | -0.1 | 0.48 | 0.83 |  | 0.16 | 0.38 | 0.67 |  | -0.002 | 0.002 | 0.33 |
| *SEC16B* | rs543874 | G |  | 0.11 | 0.23 | 0.65 |  | 0.06 | 0.34 | 0.86 |  | 0.29 | 0.6 | 0.63 |  | 0.37 | 0.47 | 0.43 |  | 0.000 | 0.003 | 0.96 |
| *TFAP2B* | rs987237 | G |  | 0.11 | 0.24 | 0.64 |  | -0.02 | 0.34 | 0.95 |  | 0.35 | 0.61 | 0.56 |  | -0.04 | 0.48 | 0.93 |  | 0.004 | 0.003 | 0.25 |
| *FAIM2* | rs7138803 | A |  | 0.07 | 0.18 | 0.7 |  | -0.05 | 0.27 | 0.86 |  | 0.29 | 0.48 | 0.54 |  | -0.03 | 0.37 | 0.93 |  | 0.003 | 0.002 | 0.2 |
| *NRXN3* | rs10150332 | C |  | 0.19 | 0.22 | 0.38 |  | 0.19 | 0.32 | 0.56 |  | 0.52 | 0.57 | 0.36 |  | 0.22 | 0.45 | 0.62 |  | 0.004 | 0.003 | 0.22 |
| *RBJ* | rs713586 | C |  | -0.18 | 0.17 | 0.29 |  | -0.31 | 0.26 | 0.23 |  | -0.74 | 0.45 | 0.1 |  | -0.33 | 0.35 | 0.36 |  | -0.004 | 0.002 | 0.08 |
| *GPRC5B* | rs12444979 | C |  | 0.09 | 0.25 | 0.71 |  | 0.21 | 0.37 | 0.56 |  | 0.2 | 0.65 | 0.76 |  | 0.24 | 0.51 | 0.64 |  | 0.000 | 0.003 | 0.99 |
| *MAP2K5* | rs2241423 | G |  | 0.13 | 0.2 | 0.51 |  | -0.04 | 0.3 | 0.9 |  | 0.71 | 0.53 | 0.18 |  | 0.43 | 0.41 | 0.3 |  | 0.003 | 0.003 | 0.26 |
| *QPCTL* | rs2287019 | C |  | 0.16 | 0.23 | 0.5 |  | 0.34 | 0.33 | 0.31 |  | 0.56 | 0.59 | 0.35 |  | 0.23 | 0.47 | 0.62 |  | 0.003 | 0.003 | 0.32 |
| *TNNI3K* | rs1514175 | A |  | 0.16 | 0.18 | 0.37 |  | 0.29 | 0.26 | 0.27 |  | 0.46 | 0.46 | 0.32 |  | 0.49 | 0.36 | 0.18 |  | 0.000 | 0.002 | 0.89 |
| *SLC39A8* | rs13107325 | T |  | -0.23 | 0.37 | 0.53 |  | -0.34 | 0.54 | 0.54 |  | 0.04 | 0.97 | 0.97 |  | -1.07 | 0.76 | 0.16 |  | 0.009 | 0.005 | 0.07 |
| *FLJ35779* | rs2112347 | T |  | 0.21 | 0.18 | 0.24 |  | 0.09 | 0.27 | 0.74 |  | 0.29 | 0.48 | 0.54 |  | 0.63 | 0.37 | 0.09 |  | -0.002 | 0.002 | 0.37 |
| *LRRN6C* | rs10968576 | G |  | 0.16 | 0.19 | 0.42 |  | 0.51 | 0.28 | 0.07 |  | 0.56 | 0.5 | 0.26 |  | 0.26 | 0.39 | 0.5 |  | 0.003 | 0.003 | 0.25 |
| *TMEM160* | rs3810291 | A |  | 0.09 | 0.19 | 0.65 |  | 0.25 | 0.27 | 0.37 |  | 0.05 | 0.48 | 0.92 |  | 0.25 | 0.38 | 0.5 |  | -0.002 | 0.002 | 0.47 |
| *FANCL* | rs887912 | T |  | 0.54 | 0.19 | **0.005** |  | 0.69 | 0.28 | **0.01** |  | 1.03 | 0.5 | **0.04** |  | 0.89 | 0.39 | **0.02** |  | 0.002 | 0.003 | 0.40 |
| *CADM2* | rs13078807 | G |  | 0.11 | 0.22 | 0.61 |  | 0.27 | 0.32 | 0.39 |  | 0.36 | 0.57 | 0.52 |  | 0.19 | 0.45 | 0.67 |  | 0.001 | 0.003 | 0.75 |
| PRKD1 | rs11847697 | T |  | 0.53 | 0.41 | 0.20 |  | 0.81 | 0.60 | 0.18 |  | 0.79 | 1.08 | 0.47 |  | 1.43 | 0.84 | 0.09 |  | -0.004 | 0.006 | 0.49 |
| *LRP1B* | rs2890652 | C |  | -0.08 | 0.23 | 0.74 |  | 0.54 | 0.33 | 0.1 |  | -0.12 | 0.59 | 0.84 |  | 0.01 | 0.47 | 0.98 |  | -0.001 | 0.003 | 0.81 |
| *PTBP2* | rs1555543 | C |  | 0.06 | 0.18 | 0.73 |  | 0.1 | 0.27 | 0.71 |  | 0.49 | 0.47 | 0.3 |  | 0.36 | 0.37 | 0.33 |  | 0.001 | 0.002 | 0.55 |
| *MTIF3* | rs4771122 | G |  | 0.37 | 0.21 | 0.07 |  | 0.45 | 0.3 | 0.13 |  | 0.74 | 0.53 | 0.16 |  | 0.84 | 0.42 | **0.04** |  | 0.000 | 0.003 | 0.88 |
| *RPL27A* | rs4929949 | C |  | 0.29 | 0.17 | 0.09 |  | 0.36 | 0.25 | 0.15 |  | 0.39 | 0.45 | 0.38 |  | 0.39 | 0.35 | 0.27 |  | 0.000 | 0.002 | 0.93 |
| *NUDT3* | rs206936 | G |  | -0.25 | 0.22 | 0.25 |  | -0.38 | 0.32 | 0.24 |  | -0.81 | 0.56 | 0.15 |  | -0.1 | 0.44 | 0.82 |  | -0.007 | 0.003 | **0.01** |
|  |  |  |  |  |  |  |  |  |  |  |  |  |  |  |  |  |  |  |  |  |  |  |

The β coefficients represent the effect sizes. EA: effect allele. SE: standard error.

*p* values were adjusted for age, gender, physical activity, smoking status and alcohol consumption. Significant p values are indicated in bold type.

**Additional file 1: Table S4.** Effect of the GPS on anthropometric variables in the MONA LISA Lille study for fully genotyped participants (n=1326).

|  |  | **β** | **SE** | **LCL** | **UCL** | ***p*** |
| --- | --- | --- | --- | --- | --- | --- |
|  | |  |  |  |  |  |
| BMI (kg/m²) | | 0.14 | 0.03 | 0.07 | 0.21 | 0.0001 |
| Body fat (%) | | 0.14 | 0.05 | 0.04 | 0.25 | 0.007 |
| Waist (cm) |  | 0.28 | 0.09 | 0.11 | 0.47 | 0.002 |
| Hip (cm) |  | 0.23 | 0.07 | 0.08 | 0.37 | 0.002 |
| Waist-to-hip ratio | | 0.0009 | 0.0004 | -0.0001 | 0.0017 | 0.08 |
|  |  |  |  |  |  |  |

The β coefficients represent the effect sizes. SE: standard error. LCL: lower confidence limit; UCL: upper confidence limit.

*p* values were adjusted for age, gender, physical activity, smoking status and alcohol consumption.

**Additional file 1: methods**

**The MONA LISA Lille study**

The participants in the MONA LISA (Monitoring National du Risque Artériel; National Monitoring of Arterial Risk) Lille study (n=1578) were recruited in 2006 and 2007 from within the Lille urban area in northern France. Subjects aged between 35 and 74 were selected randomly from electoral rolls after stratification by town size, gender and age, in order to obtain 200 participants of each gender in each 10-year age class. Details of the study's procedures and results have been reported elsewhere [1]. After providing written, informed consent, participants filled out a standard questionnaire with questions on physical activity (PA), alcohol intake, tobacco use, the participant's personal & family medical history, attitudes and knowledge concerning several diseases and any current medication use. Physical activity was assessed for the year preceding recruitment using the MONICA Optional Study of Physical Activity questionnaire [2], which had previously been assessed for validity and repeatability [3], and was previously described [4]. Briefly, the MONICA Optional Study of Physical Activity questionnaire was used to evaluate PA at work, while commuting and during recreational activities. Time spent on active commuting and work-related activities was reported and multiplied by the activity-specific energy expenditure (yielding the metabolic equivalent of task (MET)) [5]. Participants also reported their usual participation in leisure-time PA by specifying their per-week frequency and the usual duration of each session. Net PA energy expenditure in various settings was calculated in MET h/week (as described elsewhere [6]) and then categorized into quartiles as very low-intensity PA; low-intensity PA; moderate-intensity PA and high-intensity PA.

In terms of smoking exposure, participants were categorized as either smokers (i.e. individuals reporting at least one cigarette per day) or non-smokers. Ex-smokers were considered to be non-smokers. Alcohol intake was expressed as the total number of mL of alcohol per week from wine, beer, cider and spirits. Anthropometric measurements were taken by a specially trained nurse. Body weight was recorded for barefoot individuals in light clothing. The BMI was calculated according to the Quetelet equation. Obesity was considered for BMI above or equal to 30 kg/m².

After the subjects had fasted for at least 10 hours, a 20 mL blood sample was drawn into a disodium EDTA tube. The plasma glucose level was measured using a standard glucose hexokinase method (DuPont Dimension, Brussels, Belgium). Plasma insulin was measured with an enzyme immunoassay (Beckman Coulter, Villepinte, France) or an immunoradiometric assay (Immunotech, Marseille, France). The homeostatic model assessment (HOMA) scores were used as indicators of insulin resistance (HOMA-IR) and β-cell function (HOMA-B) [7,8]. Type 2 diabetes was defined according to the 1997 American Diabetes Association definition: fasting plasma glucose ≥ 7.0 mmol/l and/or treatment for diabetes, including diet and/or oral antidiabetic drugs and/or insulin [9] (n=126 individuals with T2D, n=1211 without T2D).

Blood for DNA extraction was collected in EDTA K3 tubes and sent to the Genomic Analysis Laboratory at the Institut Pasteur de Lille (Lille, France) for storage and subsequent analysis. DNA was extracted from white blood cells by using the Puregene kit (QIAGEN, Courtaboeuf, France) and stored at -20°C.

**References**

1. Goumidi L, Grechez A, Dumont J, Cottel D, Kafatos A, Moreno LA, Molnar D, Moschonis G, Gottrand F, Huybrechts I, Dallongeville J, Amouyel P, Delaunay F, Meirhaeghe A: **Impact of REV-ERB alpha gene polymorphisms on obesity phenotypes in adult and adolescent samples.** *Int J Obes (Lond)* 2013, **37:**666-672.

2. Pereira MA, FitzerGerald SJ, Gregg EW, Joswiak ML, Ryan WJ, Suminski RR, Utter AC, Zmuda JM: **A collection of Physical Activity Questionnaires for health-related research.** *Med Sci Sports Exerc* 1997, **29:**S1-205.

3. Roeykens J, Rogers R, Meeusen R, Magnus L, Borms J, de Meirleir K: **Validity and reliability in a Flemish population of the WHO-MONICA Optional Study of Physical Activity Questionnaire.** *Med Sci Sports Exerc* 1998, **30:**1071-1075.

4. Wagner A, Dallongeville J, Haas B, Ruidavets JB, Amouyel P, Ferrieres J, Simon C, Arveiler D: **Sedentary behaviour, physical activity and dietary patterns are independently associated with the metabolic syndrome.** *Diabetes Metab* 2012, **38:**428-435.

5. Ainsworth BE, Haskell WL, Leon AS, Jacobs DR, Jr., Montoye HJ, Sallis JF, Paffenbarger RS, Jr.: **Compendium of physical activities: classification of energy costs of human physical activities.** *Med Sci Sports Exerc* 1993, **25:**71-80.

6. Wagner A, Simon C, Evans A, Ferrieres J, Montaye M, Ducimetiere P, Arveiler D: **Physical activity and coronary event incidence in Northern Ireland and France: the Prospective Epidemiological Study of Myocardial Infarction (PRIME).** *Circulation* 2002, **105:**2247-2252.

7. Matthews DR, Hosker JP, Rudenski AS, Naylor BA, Treacher DF, Turner RC: **Homeostasis model assessment: insulin resistance and beta-cell function from fasting plasma glucose and insulin concentrations in man.** *Diabetologia* 1985, **28:**412-419.

8. Albareda M, Rodriguez-Espinosa J, Murugo M, de Leiva A, Corcoy R: **Assessment of insulin sensitivity and beta-cell function from measurements in the fasting state and during an oral glucose tolerance test.** *Diabetologia* 2000, **43:**1507-1511.

9. Expert Committee on the Diagnosis and Classification of Diabetes Mellitus.: **Report of the expert committee on the diagnosis and classification of diabetes mellitus.** *Diabetes Care* 2003, **26 Suppl 1:S5-20.:**S5-20.
